# Elevated remnant cholesterol and non-HDL cholesterol concentrations from real-world laboratory results: a cross-sectional study in Southeast Asians

**DOI:** 10.3389/fcvm.2024.1328618

**Published:** 2024-02-07

**Authors:** Wann Jia Loh, Heng Samuel Soh, Mon Hnin Tun, Pei Ting Tan, Chin Shern Lau, Subramaniam Tavintharan, Gerald F. Watts, Tar Choon Aw

**Affiliations:** ^1^ Department of Endocrinology, Changi General Hospital, Singapore, Singapore; ^2^ Duke-NUS Medical School, Singapore, Singapore; ^3^ Department of Cardiology, Changi General Hospital, Singapore, Singapore; ^4^ Health Services Research Unit, Changi General Hospital, Singapore, Singapore; ^5^ Clinical Trial and Research Unit, Changi General Hospital, Singapore, Singapore; ^6^ Department of Laboratory Medicine, Changi General Hospital, Singapore, Singapore; ^7^ Diabetes Centre, Admiralty Medical Centre, Singapore, Singapore; ^8^ Medical School, University of Western Australia, Perth, WA, Australia; ^9^ Department of Cardiology and Internal Medicine, Royal Perth Hospital, Perth, WA, Australia

**Keywords:** hypercholesterolaemia, LDL targets, non-HDL cholesterol, remnant cholesterol, triglyceride-rich lipoprotein, Asian ethnicity, real-world lab data, atherosclerotic cardiovascular diseases (ASCVD)

## Abstract

**Introduction:**

Triglyceride-rich remnant lipoproteins (TRLs) are considered atherogenic due to the presence of remnant cholesterol, which is transported by apolipoprotein B. In clinical practice, the concentration of TRLs can be estimated by calculating remnant cholesterol or non-HDL cholesterol levels.

**Aim:**

This study aims to investigate the proportion of patients who have low LDL cholesterol (LDL-C) concentration but elevated remnant cholesterol concentration, stratified by the presence of hypertriglyceridaemia and ethnicity, using real-world hospital data. Our secondary aim is to investigate the proportion of patients with elevated non-HDL cholesterol levels using guideline-recommended goals.

**Methods:**

A 2-year retrospective study was conducted at a single centre, analyzing lipid blood tests of all patients, including directly measured LDL-C. Fasting for blood tests was not mandatory.

**Results:**

The study included a total of 21,605 consecutive patients with plasma lipid profiles analyzed in our hospital laboratory. The median age was 61 years. In patients with ASCVD (*n* = 14,704), 23.7% had an LDL-C level of <1.8 mmol/L, 11.3% had elevated remnant cholesterol concentrations at ≥0.65 mmol/L, and 48.8% were at the non-high-density lipoprotein cholesterol (non-HDL-C) goal (<2.6 mmol/L). Among patients diagnosed with atherosclerotic cardiovascular disease (ASCVD) with LDL-C levels of <1.8 mmol/L (*n* = 3,484), only 11.9% had high levels of remnant cholesterol, but 96% of the ASCVD patients also achieved the recommended non-HDL-C target of <2.6 mmol/L. When the LDL-C level was <1.8 mmol/L, the mean concentration of remnant cholesterol was 0.214 mmol/L when the triglyceride level was <1.7 mmol/L (*n* = 3,380), vs. 0.70 mmol/L when the triglyceride level was elevated (*n* = 724), *p* < 0.001. Among patients with a triglyceride level of ≥1.7 mmol/L and an LDL-C level of <.8 mmol/L, there were 254 patients with elevated remnant cholesterol concentration and 71 patients with suboptimal non-HDL levels. Malays had a higher mean remnant cholesterol concentration compared with both Chinese and Indians across all LDL-C levels, particularly in the presence of hypertriglyceridaemia.

**Conclusions:**

An elevated remnant cholesterol concentration of >0.65 mmol/L was present in 11% of all patients. The current guideline-recommended non-HDL-C goal, which uses a 0.8 mmol/L estimate of remnant cholesterol concentration, was achieved in >92% of patients, suggesting that it is unlikely to be clinically useful for the majority of our patient population except where there is concomitant hypertriglyceridaemia. Further studies are needed to establish the appropriate non-HDL-C goal or calculated remnant cholesterol concentration, paired with the LDL-C goal or otherwise, in a Southeast Asian population.

## Introduction

Residual cardiovascular risk is present in some patients despite achieving guideline-recommended LDL cholesterol (LDL-C) targets and optimization of other cardiovascular risk factors. This may be partly related to triglyceride-rich remnant lipoproteins (TRLs) and their remnants, which are atherogenic and causal risk factors for atherosclerotic cardiovascular disease (ASCVD) (1, 2). The atherogenicity of TRL is conferred by its apolipoprotein B (apoB) content as shown by a Mendelian randomization study (2) and the failure of multiple randomized controlled trials to demonstrate cardiovascular risk reduction by lowering triglyceride levels (3–5). However, apoB testing is not routinely available in many institutions. Instead, the non-high-density lipoprotein cholesterol (non-HDL-C) is a rational cholesterol target, given that it is a summative measure of all atherogenic lipoproteins and can be calculated independently of the serum triglyceride level, and does not require fasting. This is in line with the guidelines recommending that fasting is not mandatory for cholesterol measurement, and in fact, fasting is discouraged to improve patient adherence to appointments (1, 6–11).

Recent studies have suggested that the calculated remnant cholesterol concentration is an important risk factor for ASCVD, independent of LDL-C levels, earning consideration for its use in clinical practice (12–17). A recent meta-analysis reported that having a calculated remnant cholesterol concentration of **≥**0.65 mmol/L was associated with a 1.5 times higher relative risk of ASCVD events and mortality (12, 18). Estimating TRLs, whether by non-HDL or calculated remnant cholesterol concentrations, would be helpful in informing potential residual risk attributable to atherogenic particles unrelated to LDL-C.

Following its inclusion as a secondary treatment objective by the National Cholesterol Education Programme Adult Treatment Panel III (NCEP ATP-III) two decades ago, non-HDL-C has been recommended by multiple guidelines subsequently for two purposes (6, 10, 19, 20). Firstly, non-HDL-C may be used as a tool for risk assessment particularly in patients with hypertriglyceridaemia, diabetes mellitus (DM), obesity, or very low LDL-C levels (6, 10, 19, 20). Secondly, non-HDL-C may also be used as a primary or secondary therapeutic target (6, 10, 19, 20). The intention of ATP-III was for non-HDL-C to be used as a secondary target only in patients with hypertriglyceridaemia (triglyceride level ≥2.3 mmol/L) (6, 10, 19, 20). However, more recent guidelines, such as the EAS 2019 guideline, recommend that non-HDL-C be used as a secondary target for very high-risk patients who fail to achieve LDL-C goals while acknowledging that the clinical advantages of such an approach have yet to be confirmed (6, 10, 19, 20). Large studies do support the use of non-HDL-C in the management of ASCVD; a recent risk-modelling study using Multinational Cardiovascular Risk Consortium Data from 19 countries showed that increasing the non-HDL-C concentration levels was associated with an increased 30-year cardiovascular disease (CVD) event rates (21). Other longitudinal studies suggest that non-HDL-C was a better predictor of ASCVD compared with LDL-C (22–27). In clinical practice, non-HDL-C is less favoured than other cholesterol targets. In reality, non-HDL-C is not adopted in our institution and country, where it is not automatically calculated and reported.

With the increasing number of risk factors to be measured in clinical practice, the aim of this study is to evaluate the clinical relevance to doctors by investigating the number of patients with an elevated remnant cholesterol concentration and elevated non-HDL-C in a real-world hospital setting using guideline-recommended targets, where fasting for blood tests is no longer mandatory.

## Methods

Blood lipid profiles of 21,605 consecutive patients who had blood tests analyzed in Changi General Hospital (CGH), Singapore, between 1 January 2017 and 31 May 2019 were included. CGH is a 1,000-bed hospital in eastern Singapore, the only hospital covering the east of Singapore. Lipid panels included total cholesterol concentration (TC), direct LDL-C (dLDL-C), serum triglyceride, and HDL-C. Only the first reading of the lipid panel for each patient during this period was used. The first outpatient blood lipid profile for each patient was used (*n* = 16,493, 76.3%), and if no outpatient lipid panel was available for a particular patient, the inpatient lipid panel was used instead (*n* = 5,112, 23.7%). The blood tests for lipid panels consisted of a combination of non-fasting and fasting samples because patients are not mandated to fast for lipid measurements in clinical care, although some patients and doctors may still prefer fasting (11). The non-HDL-C value was calculated by subtracting the HDL-C value from the TC value. The remnant cholesterol concentration was determined by subtracting the direct LDL-C levels from the non-HDL-C levels.

The cardiovascular risk profile data were extracted from the electronic health records using relevant keywords applied to diagnosis filters. The comorbidities included chronic kidney disease (CKD), diabetes mellitus, and atherosclerotic cardiovascular disease. The ASCVD group included patients with ICD-10 codes on electronic health records for ischaemic heart disease (IHD), coronary artery disease, myocardial infarction, ischaemic cardiomyopathy, patients requiring percutaneous coronary intervention, stroke, transient ischaemic attack, cardiovascular disease, and peripheral arterial disease. In this study, we refer to the diagnosis of IHD as patients with ICD-10 codes related to ischaemic heart disease, coronary artery disease, myocardial infarction, ischaemic cardiomyopathy, and patients requiring percutaneous coronary intervention. There is a possibility that the actual number of patients with IHD may be slightly higher than what was reported in this study because there were 2,167 patients in the electronic health record system who had a code for “cardiovascular disease” according to the ICD-10 classification, but we are unable to confirm if these patients had IHD.

In this study, the cardiovascular risk stratification and treatment goals of LDL-C paired with non-HDL-C used were as per the 2019 European dyslipidaemia guideline (28). As per the ATP-III definition, the non-HDL-C goals were set by adding 0.8 mmol/L (30 mg/dl) to LDL-C goals (28, 29). In our study, the term LDL treatment goal band refers to the four different LDL-C goals (<1.4, <1.8, <2.6, and <3.4 mmol/L) with their corresponding paired non-HDL-C goals, assuming that one of these LDL goals were used for the patient's clinical care. Because of the different goals suggested by different guidelines (e.g., LDL-C <1.4 vs. LDL-C <1.8 mmol/L for ASCVD patients) and the need for individualization of cholesterol goals depending on comorbidities, all four LDL-C treatment goal bands for each group are displayed for discussion purposes. Elevated remnant cholesterol concentration was taken as ≥0.65 mmol/L as shown by recent studies to be associated with increased ASCVD risk (12, 15).

All blood samples were centrifuged at 3,000 *g* for 5 min prior to analysis and analyzed on the Cobas c702 for TC, serum triglyceride, dLDL, and HDL within 2 h of collection, using the Roche assay. The Cobas TC, triglyceride, dLDL, and HDL assays are enzymatic colorimetric assays. The Cobas TC assay has a measuring range of 0.1–207 mmol/L and an inter-assay precision (CV) of 1.6% at TC concentrations of 2.31 and 4.85 mmol/L, respectively. The Cobas triglyceride assay has a measuring range of 0.1–50.0 mmol/L and a CV of 2.0% and 1.6% at triglyceride concentrations of 1.39 and 2.33 mmol/L, respectively. The Cobas HDL assay has a measuring range of 0.08–6.24 mmol/L and a CV of 1.5% and 0.9% at HDL concentrations of 0.88 and 1.34 mmol/L, respectively. The Cobas dLDL assay has a reported measuring range of 0.10–28.4 mmol/L and is unaffected by elevated triglyceride levels up to 23 mmol/L. The Cobas c702 dLDL assay has a CV of 1.1% at direct LDL levels of 1.51 and 2.75 mmol/L, respectively. The correlation between the non-HDL-C and dLDL-C was compared at varying triglyceride levels. Our laboratory is accredited by the College of American Pathologists, and our performance for their external quality assessment programme for lipid assays is satisfactory.

Data were presented in either mean ± standard deviation or median (interquartile range, IQR). For multivariable regression analysis, coefficient and 95% CI were reported. The covariates used for multivariable analysis were age, gender, ethnicity, the use of lipid-lowering agents (statin, fibrates, and agents of PCSK9 inhibition), and the presence of ASCVD, DM, and CKD. A two-sided *p*-value of <0.05 was considered statistically significant. Statistical analyses were performed using STATA/SE v16 software. This study was approved by our institutional review ethics board.

## Results

Table 1 summarizes the patient's demographics and blood lipid results. The predominant ethnicities in this study were Chinese, followed by Malay and Indian (61.0%, 19.6%, and 9.2%, respectively), which was similar in representation of the ethnic distribution of Singapore’s general population (74% vs. 13% vs. 9%) (30). The mean dLDL-C value was 2.70 mmol/L (SD 1.08), and the mean non-HDL-C value was 2.99 mmol/L (SD 1.18). The mean total cholesterol concentration was 4.27 mmol/L (SD 1.19). The mean serum triglyceride level was 1.59 mmol/L (SD 1.31), ranging from 0.19 to 46.51 mmol/L. Patients of Malay ethnicity had the highest mean total cholesterol concentration at 4.42 mmol/L (SD 1.30), mean dLDL-C at 2.85 mmol/L (SD 1.17), mean non-HDL-C at 3.21 mmol/L (SD 1.29), and mean triglyceride levels at 1.71 mmol/L (SD 1.59). Patients of Chinese ethnicity had the highest mean HDL-C cholesterol at 1.33 mmol/L (SD 0.42). The mean remnant cholesterol concentration was 0.29 mmol/L. When comparing patients with ASCVD (*n* = 14,704) with patients without ASCVD (*n* = 6,901), the mean remnant cholesterol concentrations were higher but the LDL-C and non-HDL concentrations were lower in patients with ASCVD compared with patients without ASCVD (remnant cholesterol 0.31 vs. 0.27 mmol/L *p* < 0.001, non-HDL 2.87 vs. 3.28 mmo/L *p* < 0.001, and LDL-C 2.56 vs. 3.01 mmol/L *p* < 0.001).

**Table 1:** The blood lipid levels of the patients and the percentage of patients at different dLDL-C, non-HDL-C, and triglyceride levels across different ethnicities. Continuous data were shown as mean (SD), whereas categorical data were presented as *n* (%). All blood lipid readings are reported in mmol/L.

	All (*n* = 21,605)	Chinese (*n* = 13,168)	Malay (*n* = 4,226)	Indian (*n* = 1,989)	Others (*n* = 2,222)
Age (years)	61.87 (39.89)	63.25 (34.75)	60.59 (47.74)	58.47 (49.12)	59.20 (42.42)
Male, *n* (%)	13,821 (63.9%)	8,470 (64.3%)	2,612 (61.8%)	1,321 (66.4%)	1,418 (63.8%)
Lipid profile					
Total cholesterol (mmol/L)	4.27 (1.19)	4.21 (1.13)	4.42 (1.30)	4.27 (1.26)	4.41 (1.30)
Direct LDL (mmol/L)	2.70 (1.08)	2.62 (1.02)	2.85 (1.17)	2.79 (1.15)	2.84 (1.14)
HDL (mmol/L)	1.28 (0.40)	1.33 (0.42)	1.20 (0.36)	1.17 (0.35)	1.23 (0.38)
Non-HDL (mmol/L)	2.99 (1.18)	2.88 (1.09)	3.21 (1.29)	3.10 (1.25)	3.19 (1.29)
Triglyceride (mmol/L)	1.59 (1.31)	1.54 (1.12)	1.71 (1.59)	1.59 (1.30)	1.69 (1.71)
Remnant cholesterol (mmol/L)	0.29 (0.51)	0.27 (0.45)	0.37 (0.59)	0.31 (0.46)	0.35 (0.67)
Comorbidities					
ASCVD, *n* (%)	14,704 (68.0%)	8,849 (67.2%)	3,028 (71.6%)	1,378 (69.2%)	1,449 (65.2%)
Diabetes mellitus, *n* (%)	8,956 (41.4%)	4,736 (35.9%)	2,183 (51.6%)	1,040 (52.2%)	997 (44.8%)
Hypertension, *n* (%)	13,059 (66.2%)	7,736 (64.8%)	2,791 (71.0%)	1,212 (65.3%)	1,320 (65.8%)
Ischaemic heart disease, *n* (%)	10,375 (52.6%)	6,029 (50.5%)	2,234 (56.8%)	1,039 (56.0%)	1,073 (53.4%)
Stroke and TIA, *n* (%)	2,976 (13.7%)	1,879 (14.2%)	588 (13.9%)	229 (11.5%)	280 (12.6%)
Peripheral arterial disease, *n* (%)	892 (4.5%)	448 (3.7%)	231 (5.8%)	107 (5.7%)	106 (5.2%)
Chronic kidney disease, *n* (%)	4,116 (20.8%)	2,282 (19.1%)	1,085 (27.6%)	316 (17.0%)	433 (21.5%)
Lipid-lowering agents					
Statins, *n* (%)	17,826 (82.5%)	10,742 (81.5%)	3,621 (85.6%)	1,668 (83.8%)	1,795 (80.7%)
Fibrates, *n* (%)	1,288 (5.9%)	764 (5.8%)	269 (6.3%)	112 (5.6%)	143 (6.4%)
PCSK9 inhibitors, *n* (%)	3 (0.01%)	1 (0.01%)	1 (0.02%)	0 (0%)	1 (0.05%)
dLDL, non-HDL and triglyceride					
dLDL-C <1.4, *n* (%)	1,489 (6.8%)	1,005 (7.6%)	250 (5.9%)	116 (5.8%)	118 (5.3%)
dLDL-C <1.8, *n* (%)	4,207 (19.4%)	2,774 (21.7%)	700 (16.5%)	385 (19.3%)	348 (15.6%)
dLDL-C <2.6, *n* (%)	11,672 (54.0%)	7,481 (56.8%)	2,050 (48.5%)	1,045 (52.5%)	1,096 (49.3%)
dLDL-C <3.4, *n* (%)	16,683 (77.2%)	10,483 (79.6%)	3,092 (73.1%)	1,483 (74.5%)	1,625 (73.1%)
Non-HDL-C <2.2, *n* (%)	5,499 (25.4%)	3,722 (28.2%)	853 (20.1%)	475 (23.8%)	449 (20.2%)
Non-HDL-C <2.6, *n* (%)	9,191 (42.5%)	6,049 (45.9%)	1,507 (35.6%)	810 (40.7%)	825 (37.1%)
Non-HDL-C <3.4, *n* (%)	15,072 (69.7%)	9,637 (73.1%)	2,680 (63.4%)	1,323 (66.5%)	1,432 (64.4%)
Non-HDL-C <4.1, *n* (%)	18,264 (84.5%)	11,489 (87.2%)	3,374 (79.8%)	1,624 (81.6%)	1,777 (79.9%)
Triglyceride <1.7, *n* (%)	14,955 (69.2%)	9,329 (70.8%)	2,759 (65.2%)	1,371 (68.9%)	1,496 (67.3%)
1.7 ≤Triglyceride <4.5, *n* (%)	6,209 (28.7%)	3,603 (27.3%)	1,362 (32.2%)	585 (29.4%)	659 (29.6%)
Triglyceride ≥4.5, *n* (%)	441 (2.0%)	236 (1.7%)	105 (2.4%)	33 (1.6%)	67 (3.0%)
Remnant cholesterol <0.65, *n* (%)	19,203 (88.9%)	11,884 (90.3%)	3,598 (85.1%)	1,784 (89.7%)	1,937 (87.2%)

ASCVD, atherosclerotic cardiovascular disease; DM, diabetes mellitus; CKD, chronic kidney disease; dLDL, directly measured low-density lipoprotein; HDL, high-density lipoprotein; TIA, transient ischaemic attack.

### Remnant cholesterol concentration

**Figure 1:**
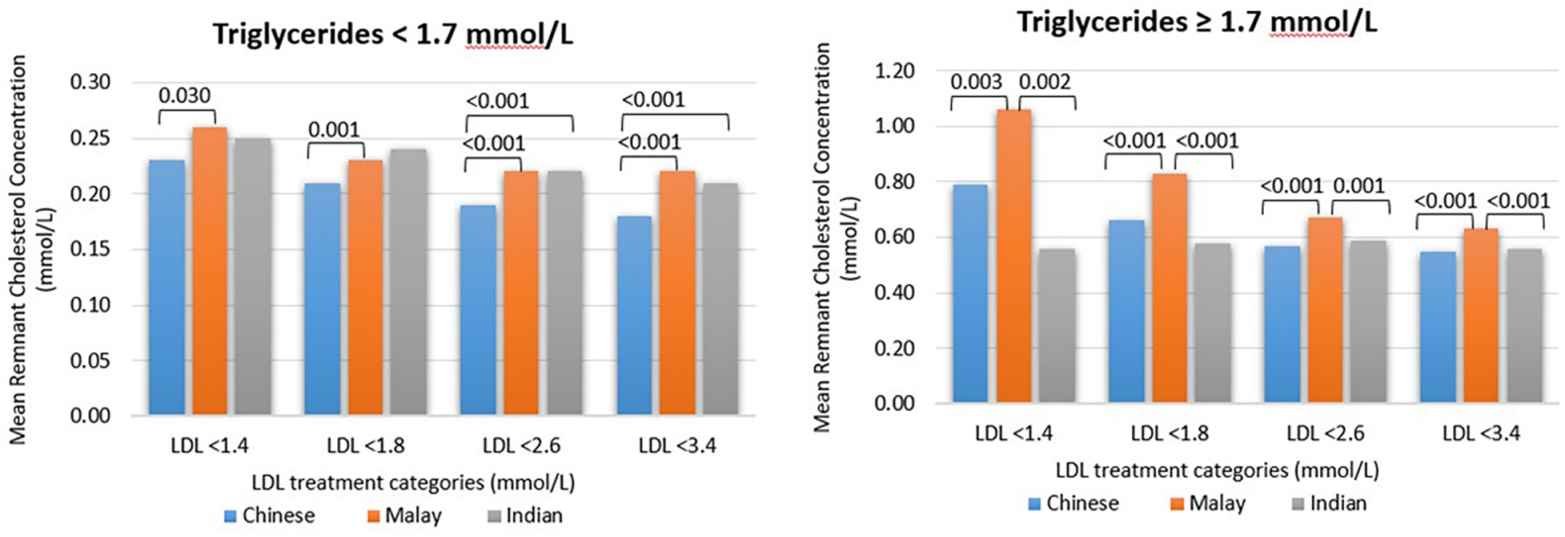
The mean remnant cholesterol concentration for every ethnicity and when the triglyceride level is <1.7 or ≥1.7 mmol/L. Patients with triglyceride levels of >4.5 mmol/L were excluded. The remnant cholesterol concentration was calculated as a difference between non-HDL-C and dLDL-C levels. The *p*-values among different ethnicity groups are shown for those with statistical significance (*p* < 0.05).

When comparing the stratification between triglyceride levels of <1.7 mmol/L and triglyceride levels of ≥1.7 mmol/L, it was found that the mean remnant cholesterol concentration was higher for all three major ethnicities when the triglyceride level was ≥1.7 mmol/L, at every LDL-C treatment goal band, *p* < 0.001 (Figure 1). For example, when the dLDL-C level was <1.4 mmol/L, comparing the triglyceride level of <1.7 mmol/L with ≥1.7 mmol/L, the mean remnant cholesterol concentration was 0.237 mmol/L (*n* = 1,206) vs. 0.859 mmol/L (*n* = 227), *p* < 0.001.

In ASCVD patients with an LDL level of <1.4 mmol/L, an elevated remnant cholesterol concentration of ≥ 0.65 mmol/L was observed in 177 patients [*n* = 101 when the TG level was ≥1.7 mmol/L]. In contrast, non-HDL-C, which uses a calculated remnant cholesterol concentration of 0.8 mmol/L (i.e., non-HDL ≥2.2 mmol/L), was present in 82 patients [*n* = 42 when the TG level was ≥1.7 mmol/L].

When the dLDL-C level was <1.8 mmol/L, comparing the triglyceride level of <1.7 mmol/L with ≥1.7 mmol/L, the mean remnant cholesterol concentration was 0.214 mmol/L (*n* = 3,380) vs. 0.70 mmol/L (*n* = 724), *p* < 0.001. When the dLDL-C level was <3.4 mmol/L, comparing the triglyceride level of <1.7 mmol/L with ≥1.7 mmol/L, the mean remnant cholesterol concentration was 0.195 mmol/L (*n* = 12,140) vs. 0.576 mmol/L (*n* = 4,239), *p* < 0.001. When the serum triglyceride level was ≥1.7 mmol/L, Malays had a higher mean remnant cholesterol concentration compared with Chinese and Indians at all LDL-C treatment goal bands (*p* < 0.05) (Figure 1). Compared with Chinese, the patients of Indian ethnicity had a higher mean remnant cholesterol concentration only when the serum triglyceride level was <1.7 mmol/L, the LDL-C level was <2.6 mmol/L, and the LDL-C level was <3.4 mmol/L, *p* < 0.001.

Among patients with ASCVD, the prevalence of elevated remnant cholesterol concentration of ≥0.65 was higher in patients with diabetes compared with those without diabetes (16.5% vs. 7.4%), with a mean cholesterol concentration of 0.39 ±** **0.49 mmol/L vs. 0.23 ± 0.42 mmol/L, respectively. Similarly, in patients with CKD, the prevalence of elevated remnant cholesterol concentration was higher in patients with diabetes compared with those without diabetes (22.3% vs. 10.7%), with a mean cholesterol concentration of 0.48 ±** **0.57 mmol/L vs. 0.27 ± 0.35 mmol/L, respectively. However, in patients with ASCVD, the prevalence of elevated remnant cholesterol concentration was similar in the group of patients on lipid-lowering agents compared with those without lipid-lowering agents (11.8% vs. 10.6%).

In a multivariable linear regression using log-transformed remnant cholesterol concentration as the dependent variable, adjusting for age, gender, ethnicity, lipid-lowering agents, and the presence of ASCVD, we found that the presence of diabetes [coefficient 0.29 (95% CI 0.25, 0.34)] and CKD [coefficient 0.26 (95% CI 0.21, 0.31)] were both independently associated with remnant cholesterol concentration, *p* < 0.001. Similarly, in a multivariable logistic regression, using a binary variable of remnant cholesterol concentration (≥0.65 vs. <0.65 mmol/L) as the dependent variable, diabetes [odds ratio 1.95 (95% CI 1.77, 2.15)] and CKD [odds ratio 2.04 (95% CI 1.83, 2.27)] were both independently associated with elevated remnant cholesterol concentration, *p* < 0.001.

### Correlation of non-HDL-C with dLDL-C in relation to triglyceride level

There was a positive correlation between non-HDL-C and dLDL-C (Supplementary Table S1, Figure S1A). The correlation of non-HDL-C and dLDL-C at different triglyceride concentrations was strongest when the triglyceride level was <4.5 mmol/L (*p*-value < 0.001). For Singaporean Chinese, there was a weak correlation between non-HDL-C and dLDL-C when the triglyceride level was ≥4.5 mmol/L, but not for Singaporean Indians and Malays (Supplementary Table S2). Table 2 shows the mean difference between non-HDL-C and LDL-C (i.e., calculated remnant cholesterol concentration) at 5 percentile increments in all patients, CKD group, DM group, and ASCVD group. In all groups, the mean difference was noticeably lower than the recommended 0.8 mmol/L, generally ranging from 0.21 to 0.61 mmol/L. The mean remnant cholesterol concentration increases at higher dLDL-C or non-HDL-C levels. The mean remnant cholesterol concentration was significantly influenced by triglyceride levels (Figure 1). When the triglyceride level was elevated, the mean remnant cholesterol concentration was nearer to 0.8 mmol/L only when the LDL level was <1.4 mmol/L; at higher LDL goal bands, the mean remnant cholesterol concentration was approximately 0.5–0.7 mmol/L.

**Table 2:** Mean remnant cholesterol distribution in all patients across different percentiles, as well as patients with ASCVD, DM, and CKD. The mean remnant cholesterol concentration is reflected in the column labelled “Difference.” All blood lipid profiles are measured in mmol/L.

	All (*n* = 21,605)			ASCVD (*n* = 14,704, 68.06%)			DM (*n* = 8,956, 41.45%)			CKD (*n* = 4,116, 20.87%)		
Study cohort percentile	dLDL-C	Non-HDL-C	Difference	dLDL-C	Non-HDL-C	Difference	dLDL-C	Non-HDL-C	Difference	dLDL-C	Non-HDL-C	Difference
5	1.30	1.51	0.21	1.24	1.44	0.20	1.21	1.49	0.28	1.22	1.47	0.25
10	1.53	1.75	0.22	1.46	1.67	0.21	1.44	1.72	0.28	1.46	1.73	0.27
15	1.70	1.91	0.21	1.61	1.82	0.21	1.60	1.88	0.28	1.61	1.90	0.29
20	1.82	2.05	0.23	1.73	1.95	0.22	1.73	2.01	0.28	1.76	2.04	0.28
25	1.94	2.19	0.25	1.83	2.07	0.24	1.84	2.15	0.30	1.88	2.19	0.31
30	2.05	2.31	0.26	1.93	2.19	0.26	1.94	2.26	0.32	2.00	2.31	0.31
35	2.16	2.42	0.26	2.03	2.30	0.27	2.04	2.38	0.34	2.11	2.44	0.33
40	2.27	2.54	0.27	2.13	2.41	0.28	2.14	2.48	0.34	2.23	2.56	0.33
45	2.39	2.66	0.27	2.23	2.52	0.29	2.24	2.59	0.35	2.35	2.68	0.33
50	2.51	2.79	0.28	2.33	2.63	0.30	2.35	2.71	0.36	2.47	2.81	0.34
55	2.63	2.92	0.29	2.44	2.76	0.32	2.46	2.84	0.38	2.59	2.95	0.36
60	2.77	3.07	0.30	2.57	2.89	0.32	2.58	2.98	0.40	2.73	3.09	0.36
65	2.92	3.23	0.31	2.70	3.04	0.34	2.72	3.14	0.42	2.89	3.24	0.35
70	3.10	3.41	0.31	2.87	3.22	0.35	2.89	3.32	0.43	3.06	3.42	0.36
75	3.30	3.61	0.31	3.07	3.42	0.35	3.08	3.52	0.44	3.23	3.59	0.36
80	3.52	3.85	0.33	3.31	3.66	0.35	3.31	3.76	0.45	3.44	3.82	0.38
85	3.81	4.13	0.32	3.60	3.98	0.38	3.60	4.10	0.50	3.69	4.10	0.41
90	4.16	4.52	0.36	4.00	4.39	0.39	3.99	4.53	0.54	4.04	4.49	0.45
95	4.70	5.09	0.39	4.59	5.02	0.43	4.57	5.18	0.61	4.63	5.11	0.48

ASCVD, atherosclerotic cardiovascular disease; DM, diabetes mellitus; CKD, chronic kidney disease; dLDL, directly measured low-density lipoprotein; HDL, high-density lipoprotein.

For this analysis, patients with triglyceride levels of ≥4.5 mmol/L were excluded because of the weaker correlation between non-HDL-C and LDL-C when the triglyceride level was ≥4.5 mmol/L (Supplementary Figure S1B).

### Direct LDL-C and non-HDL-C levels in patients

Among all the patients, 6.8% had LDL-C levels of <1.4 mmol/L, 19.4% had LDL-C levels of <1.8 mmol/L, 54.0% had LDL-C levels of <2.6 mmol/L, and 77.2% had LDL-C levels of <3.4 mmol/L (Table 1). A similar distribution pattern of the number of patients within each LDL treatment target band was observed within each ethnicity subgroup. The percentage of patients of Chinese, Malay, and Indian ethnicity with non-HDL-C levels of <2.2 mmol/L was slightly more variable at 28.2%, 20.1%, and 23.8%, respectively. The percentage of patients of Chinese, Malay, and Indian ethnicity with non-HDL-C levels of <4.1 mmol/L was 87.2%, 79.8%, and 81.6%, respectively. The majority of patients (85%–90%) had a remnant cholesterol concentration of <0.65 mmol/L.

Table 3 shows the percentage of patients with ASCVD, DM, and CKD diagnoses achieving different LDL-C, non-HDL-C, or the paired LDL-C and non-HDL-C treatment goals. More patients achieve favourable non-HDL-C than LDL-C, whichever LDL-C treatment goal band was adopted. For example, more patients had non-HDL-C levels of <2.6 mmol/L than LDL-C levels of <1.8 mmol/L (42.5% vs. 19.4%) in all patients, as well as within the ASCVD group (48.8% vs. 23.7%), DM group (45.3% vs. 23.5%), and CKD group (42.0% vs. 21.9%). Similarly, more patients had non-HDL-C levels of <2.2 mmol/L than LDL-C levels of <1.4 mmol/L in the ASCVD group (30.3% vs. 8.6%) and DM group (27.1% vs. 8.9%).

**Table 3:** Percentage of patients with ASCVD, DM, and CKD that achieved dLDL-C target alone, non-HDL-C target alone, or both the dLDL-C and the paired non-HDL-C within each treatment target band. All lipid profiles are reported in mmol/L.

	ASCVD (*n* = 14,704)	DM (*n* = 8,956)	CKD (*n* = 4,116)	Remnant cholesterol <0.65 (*n* = 19,203)
dLDL-C <1.4	8.6%	8.9%	8.6%	6.5%
dLDL-C <1.8	23.7%	23.5%	21.9%	19.4%
dLDL-C <2.6	61.4%	60.9%	55.6%	54.6%
Non-HDL-C <2.2	30.3%	27.1%	25.5%	27.9%
Non-HDL-C <2.6	48.8%	45.3%	42.0%	46.1%
Non-HDL-C <3.4	74.5%	72.0%	69.4%	73.4%
dLDL-C <1.4 & non-HDL-C <2.2	8.0%	7.9%	11.4%	6.5%
dLDL-C <1.8 & non-HDL-C <2.6	22.8%	22.1%	30.1%	19.4%
dLDL-C <2.6 & non-HDL-C <3.4	60.3%	59.0%	78.6%	54.6%
Remnant cholesterol ≥0.65	11.7%	16.3%	19.3%	

Table 4 shows that amongst patients in every LDL-C treatment goal band, 92.4%–98.4% of patients had a corresponding non-HDL-C within the paired band. Amongst patients with LDL-C levels of <1.8 mmol/L, 95.7% of these patients had a paired non-HDL-C level of <2.6 mmol/L. However, the converse was not true, as only 43.8% of patients with non-HDL-C levels of <2.6 mmol/L had the paired LDL-C within the same goal band. Within the non-HDL-C goal band of <2.2 mmol/L, only 25% of patients who met this goal achieved the corresponding paired LDL-C goal of <1.4 mmol/L. These findings were similarly noted in subgroup analyses of patients with high and very high ASCVD risk groups (CKD, DM, established ASCVD). Within the group with established ASCVD, 96.1% (*n* = 3,345) of patients had LDL-C levels of <1.8 mmol/L, and 46.7% of patients had non-HDL-C levels of <2.6 mmol/L. In the group of patients with DM, 93.9% (*n* = 1,980) of patients had LDL-C levels of <1.8 mmol/L, and 48.8% of patients had non-HDL-C levels of <2.6 mmol/L. In the group of patients with CKD, 94.3% (*n* = 1,238) of patients had LDL-C levels of <1.8 mmol/L, and 49.2% of patients had non-HDL levels of <2.6 mmol/L.

**Table 4:** Percentage of patients that would have achieved the corresponding non-HDL-C or LDL-C if either of these were used as primary targets.

	All (*n* = 21,605)		ASCVD (*n* = 14,704, 68.06%)		DM (*n* = 8,956, 41.45%)		CKD (*n* = 4,116, 20.87%)	
	% patients who met LDL-C target that also achieved non-HDL- C target	% of patients who met non-HDL-C target that also achieved LDL-C target	% patients who met LDL-C target that also achieved non-HDL- C target	% of patients who met non-HDL-C target that also achieved LDL-C target	% patients who met LDL-C target that also achieved non-HDL- C target	% of patients who met non-HDL-C target that also achieved LDL-C target	% patients who met LDL-C target that also achieved non-HDL- C target	% of patients who met non-HDL-C target that also achieved LDL-C target
dLDL-C <1.4 & non-HDL-C <2.2	92.4	25.0	93.7	26.2	88.8	29.3	90.5	30.7
dLDL-C <1.8 & non-HDL-C <2.6	95.7	43.8	96.1	46.7	93.9	48.8	94.3	49.2
dLDL-C <2.6 & non-HDL-C <3.4	97.9	75.8	98.2	81.3	96.9	81.9	97.2	77.8
dLDL-C <3.4 & non-HDL-C <4.1	98.4	89.8	98.7	93.1	97.5	93.7	97.7	90.6
	% patients who met LDL-C target that also achieved RC <0.65 mmol/L	% of patients who had RC <0.65 mmol/L that also achieved LDL-C target	% patients who met LDL-C target that also achieved RC <0.65 mmol/L	% of patients who had RC <0.65 mmol/L that also achieved LDL-C target	% patients who met LDL-C target that also achieved RC <0.65 mmol/L	% of patients who had RC <0.65 mmol/L that also achieved LDL-C target	% patients who met LDL-C target that also achieved RC <0.65 mmol/L	% of patients who had RC <0.65 mmol/L that also achieved LDL-C target
dLDL-C <1.4 & RC <0.65	84.2	6.5	85.9	8.3	79.7	8.5	80.1	8.2
dLDL-C <1.8 and RC <0.65	88.3	19.4	89.1	23.9	84.7	23.8	84.5	21.9

ASCVD, atherosclerotic cardiovascular disease; DM, diabetes mellitus; CKD, chronic kidney disease; dLDL, directly measured low-density lipoprotein; HDL, high-density lipoprotein; RC, remnant cholesterol concentration.

There were 138 patients with ASCVD who had low LDL-C levels of <1.8 mmol/L but elevated non-HDL-C levels of ≥2.6 mmol/L; only 1.5% of these patients had triglyceride levels of <1.7 mmol/L, whereas 47.1% had triglyceride ≥4.5 mmol/L (Supplementary Table S3). There were 82 patients with ASCVD with low LDL-C levels <1.4 mmol/L, but elevated non-HDL-C levels of ≥2.2 mmol/L; of these, only 6.1% had normal triglyceride levels of <1.7 mmol/L, whereas 42.7% had triglyceride levels of ≥4.5 mmol/L (Supplementary Table S4).

In patients with ASCVD, when the triglyceride level was ≥1.7 mmol/L, there were 71 patients with LDL-C levels of <1.8 mmol/L and non-HDL-C levels of ≥2.6 mmol/L vs. 254 patients with both LDL levels of <1.8 and a remnant cholesterol concentration of ≥0.65 mmol/L. When the triglyceride level was <1.7 mmol/L, there were only two patients in the ASCVD group with both LDL-C levels of <1.8 mmol/L and non-HDL-C levels of ≥2.6 mmol/L, vs. 55 patients with both LDL levels of <1.8 and a remnant cholesterol concentration of ≥0.65 mmol/L (Supplementary Table S5). When the triglyceride level was ≥1.7 mmol/L, there were 42 patients with LDL-C levels of <1.4 mmol/L and non-HDL-C levels of ≥2.2 mmol/L, as opposed to 101 patients in the ASCVD group with both LDL-C levels of <1.4 but an elevated remnant cholesterol concentration of ≥0.65 mmol/L (Supplementary Table S5).

Our analyses showed that in patients with hypertriglyceridaemia (≥1.7 mmol/L), there were 2,482 (39.9%) individuals with a low HDL level of <1 mmol/L. There was a high prevalence (73.3%) of dyslipidaemia patterns of both hypertriglyceridaemia (TG ≥1.7 mmol/L) and low HDL cholesterol concentration (HDL <1 mmol/L) in patients with ASCVD. We report that the elevated remnant cholesterol concentration of ≥0.65 mmol/L affected more individuals with both hypertriglyceridaemia and low HDL cholesterol concentration than individuals with hypertriglyceridaemia but normal HDL cholesterol concentration of ≥1 mmol/L: 45.4% (with a mean RC of 0.69 ± 0.35 mmol/L) vs. 18.5% (with a mean RC of 0.45 ± 0.27 mmol/L), respectively.

## Discussion

Our study showed that elevated remnant cholesterol concentration affected approximately 11% of patients with ASCVD and 16% of patients with diabetes. These patients may potentially benefit from lipid-lowering agents aiming to reduce residual risk such as with icosapent ethyl (31). However, the majority of patients (92%–96%) who achieved a low LDL-C level had also achieved the guideline-recommended HDL goal, regardless of ethnicity or cardiovascular comorbidities. This suggests that adopting the ATP-III-defined non-HDL-C goal as a secondary target would not be clinically useful in our study cohort of patients at high risk for ASCVD, except in the less common instance when the triglyceride level was concomitantly severely elevated. In addition to the vast number of studies on LDL and non-HDL (6, 10, 19, 20), a recent analysis of prospective studies and Mendelian randomization studies has found that elevated remnant cholesterol concentration is an independent causal factor associated with ASCVD risk, suggesting a role in clinical practice (12–17). However, with the increasing suggestions of the use of multiple clinical risk factors and lipid markers, more efforts are necessary to rationalize the lipid goals in clinical practice. Our study suggests that, at least cross-sectionally, targeting LDL goals as a first-line goal still appears to be strategic.

Our analysis suggests that the non-HDL-C goals need to be revised to appropriately reflect the residual risk of TRLs. Because the calculated mean difference between LDL and non-HDL in our cohort was lower than 0.8 mmol/L, the use of remnant cholesterol concentration of ≥0.65 mmol/L would identify more patients with residual risk although the use of either marker appears to be relevant in only a small subset of patients. However, the mean remnant cholesterol concentration differed according to ethnicity, the presence of diabetes, and CKD and was dependent on triglyceride levels, potentially affected by the presence of a low HDL cholesterol concentration. Supported by recent studies, our findings suggest that there is a need for individualization of non-HDL-C goals and reference ranges for patients of different ethnicities, as well as potentially considering other factors, such as triglyceride levels (32–34).

A large study of 73,495 Chinese patients also found that more patients achieved the non-HDL-C level of <2.6 mmol/L (100 mg/dl) than the LDL-C level of <1.8 mmol/L (70 mg/dl) at 39.4% vs. 27.2%, respectively (33). The authors suggested that to ascertain the corresponding appropriate non-HDL-C goal, 20–25 mg/dl should be added to LDL-C levels depending on the serum triglyceride level, instead of adding 0.8 mmol/L (30 mg/dl) to LDL-C regardless of serum triglyceride levels (33). This is because in patients with LDL-C levels of ≤2.6 mmol/L, the mean difference between the LDL-C and non-HDL-C was influenced by the level of triglyceride; a difference of 0.21 mmol/L (19.1 mg/dl) in patients with TG levels of ≤1.7 mmol/L and 0.28 mmol/L (24.6 mg/dl) in patients with TG levels of >1.7 mmol/L (33). A large study of approximately 15,000 patients in Brazil also showed that non-HDL-C was up to 8 mg/dl lower than the guideline goals, with a significant treatment category discordance between LDL-C and non-HDL-C, particularly for those with low LDL-C (<2.6 mmol/L) and high triglyceride levels (≥1.7 mmol/L) (34). In our study, we showed that the mean difference between non-HDL-C and dLDL-C was approximately 2 times higher when the triglyceride level was ≥1.7 mmol/L, suggesting the presence of an increased amount of remnant cholesterol concentration (i.e., non-LDL proatherogenic particles). These observations bring into question the derivation of the formula for adding of 30 mg/dl (≈0.8 mmol/L) to LDL-C to calculate the non-HDL-C goal, as recommended by the NCEP ATP-III guideline. The reason adopted by the ATP-III guideline was that a VLDL-C level of <30 mg/dl was likely normal, and the increased cardiovascular risk was seen when the VLDL-C level was >30 mg/dl (29). However, the NCEP ATP-III guideline recommends using non-HDL-C goals as secondary targets for patients with triglyceride levels of ≥2.2 mmol/L (200 mg/dl) only (35). The majority of the current lipid guidelines, such as the AHA/ACC/NLA 2018 (7), ESC 2019 (1), AACE 2020 (8), JAS 2017 (9), and NICE guidelines (19), recommend the ATP-III-recommended non-HDL-C goals. An exception is the 2021 Canadian Cardiovascular Society Guidelines, which recommend corresponding non-HDL-C goals based on percentile equivalents of LDL-C rather than a fixed difference of 0.8 mmol/L (10); at the LDL-C goal of <1.8 mmol/L, the corresponding non-HDL-C goal is <2.4 mmol/L. Some guidelines, such as NLA (2015), NICE, and the Canadian Cardiovascular Society (2021) guidelines, favour the use of non-HDL-C over LDL-C because of the limitations of using the calculated LDL-C (cLDL) using the Friedewald formula (6, 10, 19, 20).

We report that non-HDL-C strongly correlated with LDL-C when the triglyceride level was <4.5 mmol/L, with LDL-C accounting for 95% of the variability of non-HDL-C since the majority of the atherogenicity of non-HDL-C is made up of LDL-C. Unsurprisingly, they provide a very similar ASCVD risk, particularly when the triglyceride levels are low (21, 28, 36). However, at higher triglyceride levels, there is a greater discordance between non-HDL-C and LDL-C (36, 37), which may affect ASCVD risk. In the SHEP study cohort, LDL-C was an independent predictor of ASCVD if serum triglyceride levels were <4.52 mmol/L, but non-HDL-C was an independent predictor regardless of the triglyceride level (38). The MESA study showed that there was 44% discordance between LDL-P measured by nuclear magnetic resonance (NMR) spectroscopy when compared with non-HDL-C (37). This discordance was affected by serum triglycerides, insulin resistance, ethnicity, and medications. Non-HDL-C, being a summation of all atherogenic proteins, including triglyceride-rich remnant lipoproteins and lipoprotein (a) [Lp(a)] cholesterol, individually with an established atherogenicity effect, is useful to provide additional information on non-LDL atherogenic lipoproteins (1, 39). As per our study findings, the role of the non-HDL goal at the currently recommended thresholds appears to be mainly confined to cases of hypertriglyceridaemia, while LDL-C concentration may be insufficient as a marker of TRLs. Further studies are needed to evaluate whether non-HDL-C or remnant cholesterol concentration is more useful in clinical practice.

The sub-analysis of our findings showed that there is a mild difference between the correlation of non-HDL-C and dLDL-C in different ethnicities. For Singaporean Chinese, there is still a weak correlation between these two variables when the triglyceride level is ≥4.5 mmol/L, but not for Singaporean Indians and Malays. When the triglyceride level was ≥1.7 mmol/L, the calculated remnant cholesterol concentration was highest in Malay, followed by Indian and Chinese ethnicities, suggesting that Malays had higher concentrations of proatherogenic particles that were not LDL-C in our study. However, the number of patients is too small for further discrimination based on higher triglyceride levels. In the MESA study, Hispanic ethnicity was associated with having LDL-P > non-HDL discordance (37). Lp(a), which is predominantly determined by genetics, is noted to be higher in Indians, followed by Malays and Chinese (40).

A study using NHANES data by Kilgore et al. showed a discrepancy between using LDL-C and non-HDL-C for CVD risk classification (41). Out of 4,986 subjects, 15.7% had high LDL-C with a normal non-HDL-C compared with only 9.7% having a normal LDL-C with a high non-HDL-C (41). Thus, relying on non-HDL-C as a single measure for cardiovascular risk runs a higher risk of misclassification than a single measure of LDL-C as shown in our study. In individuals with type 2 diabetes mellitus (T2DM), there is often combined dyslipidaemia, characterized by reduced HDL, increased LDL, increased triglyceride, and elevated TRLs, which could explain the slight difference in the mean calculated remnant cholesterol concentration in the T2DM group compared with the CVD or CKD groups. Our findings of non-uniform discrepancies between LDL-C and non-HDL-C across different percentiles in different risk groups support the need for further studies to determine the appropriate non-HDL-C goal that corresponds to each LDL-C goal, specifically the elevated remnant cholesterol concentration (32–34). Studies are needed to confirm whether guideline-defined non-HDL targets or a general threshold for remnant cholesterol concentration (e.g., ≥0.65 mmol/L) should be used in clinical practice.

One limitation of our study is that it is cross-sectional, without information regarding clinical outcomes or circumstances (e.g., sepsis), and lacks sufficient data on body mass index (missing data >50%). We cannot determine the proportion of patients who self-opted to fast for blood lipid measurement despite being informed that it is not mandatory according to the guidelines. However, this reflects a real-world situation in which certain patients and healthcare professionals continue to prefer fasting blood test results (19, 28). Only one lipid reading per patient was used, and hence, this study was not aimed to discuss the reasons for low rates of achievement of cholesterol goals. Our study did not have sufficient numbers of measurements of other lipoproteins such as VLDL cholesterol, apoB, and Lp(a) concentrations. The prescription of omega-3 fish oil supplementation was not captured in our dataset because of the low prescription rates in our local setting and likely low adherence rates related to tolerability (42). The discussions in this study serve only to generate hypotheses, and further larger studies are needed to establish the effect of hypertriglyceridaemia and other lipoprotein remnants on non-HDL-C and LDL-C, as well as to establish the clinical significance of this in our multi-ethnic population. Another limitation of our study is that we have a relatively small number of patients with triglyceride levels of >5 mmol/L, despite the initial large sample size of the cohort. This is not surprising due to the remarkably low prevalence rate of severe hypertriglyceridaemia at 1.7% for triglyceride levels of >5.7 mmol/L (>500 mg/dl), as observed in a study conducted on children in the United States (43). Similarly, in the Norwegian population, only 0.1% had severe triglyceride levels of >10 mmol/L (44). The major strength of this study is the use of directly measured LDL-C using a robust immunoassay in a large sample size with various ethnicities.

In conclusion, elevated remnant cholesterol concentration using the threshold of ≥0.65 mmol/L affects approximately 11% of the patients in real-world situations. The current guidelines recommend that setting non-HDL-C goals in addition to LDL-C goals is unlikely to provide additional benefit, except in patients with severe hypertriglyceridaemia. Further studies are needed to establish the appropriate non-HDL-C goal or calculated remnant cholesterol concentration, in combination with the LDL-C goal or otherwise, for different cardiovascular risk groups amongst different ethnicities. It is also necessary to investigate whether this would provide additional advantages in measuring patients who have already achieved low LDL-C levels.

## Data Availability

The original contributions presented in the study are included in the article/Supplementary Material, further inquiries can be directed to the corresponding author.
