# Supplementary material for: Elevated remnant cholesterol and non-HDL cholesterol concentrations from real-world laboratory results: a cross-sectional study in Southeast Asians
Source: Front Cardiovasc Med. 2024 Feb 7;11:1328618. doi: 10.3389/fcvm.2024.1328618 (PMC10879277; doi:10.3389/fcvm.2024.1328618)
Supplement: Supplementary file 1 [file Datasheet1.pdf]

Supplementary Figure 1a: Correlation between non-HDL-C with dLDL-C

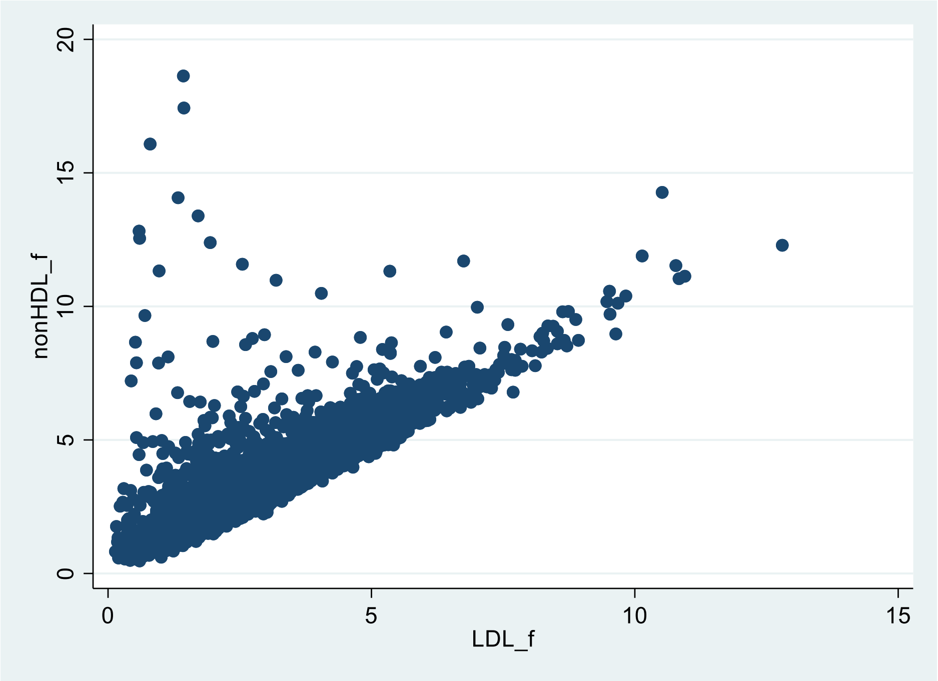

Supplementary Figure 1b: Correlation between non-HDL-C with dLDL-C when triglyceride ≥ 4.5 mmol/L

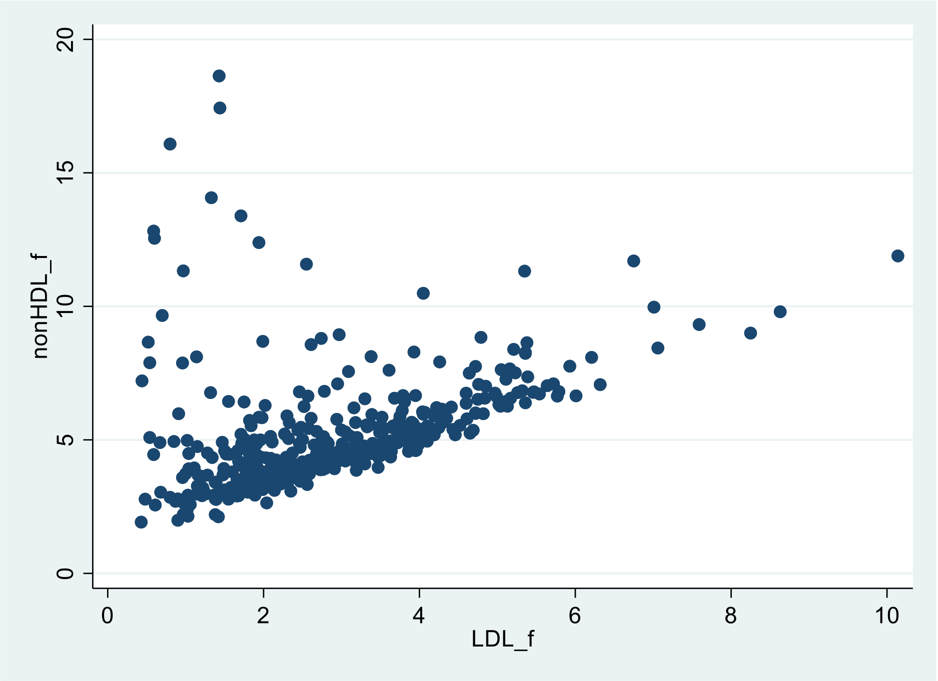

Supplementary Table 1: Association between non-HDL-C with direct LDL-C at different triglyceride concentrations

(*Univariable analysis and multivariable analysis are shown. Multivariable analysis accounted for age, sex and race. Coefficient(β), 95% confidence interval(CI) and p value are shown.)*

		Univariable			Multivariable		
Triglyceride level (mmol/L)	n	β	95% CI	p value	β	95% CI	p value
<0.5	242	0.898	0.869-0.927	<0.001	0.902	0.872-0.932	<0.001
0.5-<1	5739	0.894	0.886-0.903	<0.001	0.894	0.886-0.902	<0.001
1 to <1.5	7272	0.889	0.882-0.897	<0.001	0.887	0.879-0.896	<0.001
1.5 to <2	4051	0.887	0.876-0.899	<0.001	0.887	0.876-0.898	<0.001
2 to <2.5	1967	0.899	0.884-0.914	<0.001	0.897	0.881-0.912	<0.001
2.5 to <3	945	0.914	0.892-0.935	<0.001	0.910	0.888-0.932	<0.001
3 to <3.5	541	0.947	0.916-0.978	<0.001	0.946	0.915-0.977	<0.001
3.5 to <4	285	0.887	0.839-0.935	<0.001	0.878	0.829-0.928	<0.001
4 to <4.5	173	0.876	0.816-0.936	<0.001	0.865	0.804-0.927	<0.001
4.5 to <5	102	1.053	0.954-1.152	<0.001	1.054	0.954-1.154	<0.001
5 to <5.5	66	0.833	0.685-0.980	<0.001	0.825	0.661-0.988	<0.001
≥4.5	370	0.735	0.599-0.870	0.001	0.728	0.592-0.863	<0.001
≥5	268	0.629	0.451-0.809	<0.001	0.623	0.442-0.804	<0.001
5 to <10	230	0.859	0.759-0.958	<0.001	0.851	0.750-0.952	<0.001
≥10	38	0.183	(-)0.601-0.967	0.638	-0.002	(-)0.874-0.869	0.996

Supplementary Table 2: Subgroup analysis in different ethnicities to assess correlation between non-HDL-C and direct LDL-C at different triglyceride levels

	**Chinese**				**Malay**				**Indian**				**Others**			
Triglyceride Level(mmol/L)	n	β	95%CI	p value	n	β	95%CI	p value	n	β	95%CI	p value	n	β	95%CI	p value
0 to <3	12405	0.92	0.914-0.927	<0.001	3886	0.944	0.932-0.956	<0.001	1875	0.923	0.907-0.940	<0.001	2050	0.931	0.915-0.946	<0.001
3 to <3.5	297	0.963	0.922-1.004	<0.001	137	0.928	0.860-0.997	<0.001	48	0.939	0.852-1.026	<0.001	59	0.904	0.819-0.987	<0.001
3.5 to <4	172	0.94	0.881-0.999	<0.001	62	0.775	0.660-0.889	<0.001	17	1.026	0.798-1.255	<0.001	34	0.841	0.705-0.976	<0.001
4 to <4.5	92	0.808	0.713-0.903	<0.001	48	0.878	0.762-0.994	<0.001	13	0.971	0.709-1.233	<0.001	20	0.903	0.778-1.028	<0.001
≥4.5	192	0.765	0.644-0.888	<0.001	90	0.591	0.223-0.959	0.002	32	0.519	(-)0.073-1.113	0.083	56	0.778	0.438-1.117	<0.001
4.5 to 5.5	90	0.878	0.774-0.983	<0.001	43	1.186	0.994-1.376	<0.001	11	0.849	0.700-0.997	<0.001	24	0.852	0.619-1.085	<0.001

Supplementary Table 3: Characteristics of patients with both LDL-C <1.8 mmol/L and non-HDL-C ≥2.6 mmol/L levels in patients with ASCVD (n=138).

	**All** (n=138) n (%), Mean (SD)	**Chinese** (n=73) n (%), Mean (SD)	**Malay** (n=41) n (%), Mean (SD)	**Indian** (n=7) n (%), Mean (SD)	**Others** (n=17) n (%), Mean (SD)
Age (years)	59.51 (12.50)	61.32 (12.06)	58.42 (13.26)	53.86 (9.44)	56.76 (13.06)
Male (%)	101 (73.1%)	51 (69.8%)	30 (73.1%)	6 (85.7%)	14 (82.3%)
Comorbidities					
Ischemic heart disease (IHD)	101 (73.1%)	54 (73.9%)	30 (73.1%)	6 (85.7%)	11 (64.7%)
Hypertension	113 (81.8%)	61 (83.5%)	31 (75.6%)	6 (85.7%)	15 (88.2%)
Diabetes Mellitus (DM)	97 (70.2%)	51 (69.8%)	29 (70.7%)	6 (85.7%)	11 (64.7%)
Stroke/Cerebrovascular Disease	28 (20.2%)	16 (21.9%)	6 (14.6%)	2 (28.5%)	4 (23.5%)
Peripheral Arterial Disease (PAD)	17 (12.3%)	6 (8.2%)	6 (14.6%)	2 (28.5%)	3 (17.6%)
Chronic Kidney Disease (CKD)	64 (46.3%)	32 (43.8%)	21 (51.2%)	3 (42.8%)	8 (47.1%)
Anti-lipid Medication Profile					
Statins	119 (86.2%)	62 (84.9%)	35 (85.3%)	6 (85.7%)	16 (94.1%)
Fibrates	43 (31.1%)	27 (36.9%)	9 (21.9%)	5 (71.4%)	2 (11.7%)
PCSK9 Inhibitors	0 (0%)	0 (0%)	0 (0%)	0 (0%)	0 (0%)
Triglyceride Level (mmol/L)					
TG <1.7	2 (1.5%)	0 (0%)	1 (2.4%)	0 (0%)	1 (5.8%)
1.7 < TG < 4.5	71 (51.4%)	39 (53.4%)	24 (58.5%)	2 (28.5%)	6 (35.2%)
TG ≥ 4.5	65 (47.1%)	34 (46.5%)	16 (39.0%)	5 (71.4%)	10 (58.8%)

TG: triglyceride concentration

<u>Supplementary Table 4:</u> Characteristics of patients with both LDL-C<1.4mmol/L and non-HDL-C≥2.2 mmol/L levels (n=82).

Supplementary Table 5: Number of patients with both low direct LDL-C and elevated non-HDL or elevated remnant cholesterol concentration (RC), stratified by triglyceride concentration (TG) . All units are in mmol/L

	TG ≥1.7 (n=4214)	TG <1.7 (n=10,213)
dLDL-C <1.8 & non-HDL-C≥2.6	71	2
dLDL-C <1.8 & RC ≥0.65	254	55
dLDL-C <1.4 & non-HDL-C≥2.2	42	5
dLDL-C <1.4 & RC ≥0.65	101	38
